# 149 Accelerometer-based measurement of physical activity and sleep duration in Denmark: A Large Population-Based Study

**DOI:** 10.1093/eurpub/ckae114.038

**Published:** 2024-09-26

**Authors:** Hannah Ahrensberg, Martin Eghøj, Ola Ekholm, Anne Illemann Christensen, Christina Bjørk Petersen, Mette Rasmussen

**Affiliations:** National Institute of Public Health, University of Southern Denmark, Studiestræde 6, 1455 Denmark, Denmark; National Institute of Public Health, University of Southern Denmark, Studiestræde 6, 1455 Denmark, Denmark; National Institute of Public Health, University of Southern Denmark, Studiestræde 6, 1455 Denmark, Denmark; National Institute of Public Health, University of Southern Denmark, Studiestræde 6, 1455 Denmark, Denmark; National Institute of Public Health, University of Southern Denmark, Studiestræde 6, 1455 Denmark, Denmark; National Institute of Public Health, University of Southern Denmark, Studiestræde 6, 1455 Denmark, Denmark

## Abstract

**Purpose:**

Public health surveillance involves regular assessment of health indicators, including physical activity, sedentary behavior, and sleep. Traditionally, self-reported measures have been used, but recent advancements in accelerometer technology and administration offer a new approach. The aim of this study is to monitor and explore accelerometer-based measurements of physical activity and sleep duration in in the adult Danish population.

**Methods:**

Data were obtained from the Danish National Health Survey conducted in 2023. From participants delivering a web-based response, 6,993 individuals (age 16 to 92 years) were invited to participate in an accelerometer-based measurement of physical activity, sedentary behavior, and sleep. A total of 1,595 individuals accepted (22,8%). Each participant received a SENS motion® accelerometer in the post, an instruction letter (with a QR-code to access a mobile-application), a mounting patch, and a pre-paid return envelope. Data were analyzed using the MOTUS algorithm, which is a refined version of the Acti4-algoritmen.

**Results:**

The average time spent on physical activity with moderate to vigorous intensity was 31 min/day (Range: 0.5-178). Overall, 58% were sufficiently physically active according to WHO recommendations. The average total sedentary time was 527 min/day (equivalent to 8 hours and 47 minutes). Men were more sedentary than women. Participants took an average of 10,577 steps/day and cycled for 19 min/day (median: 13 minutes; range: 3-103 min/day). Within the primary sleep period, the average sleep time was 7 hours and 13 minutes/day and 33% of participants slept less than 7 hours/day.

**Conclusion:**

The use of accelerometers has the potential to contribute significantly to monitoring of physical activity, sedentary behavior, and sleep at the population level in Denmark. However, this should be combined with questionnaire-based information as self-report and accelerometer measurements does not seem to capture the same facets of physical activity. Nevertheless, further development is necessary to enhance administration and user experiences.

